# The role of Syk/CARD9 coupled C-type lectins in antifungal immunity

**DOI:** 10.1002/eji.201041252

**Published:** 2010-12-10

**Authors:** Rebecca A Drummond, Shinobu Saijo, Yoichiro Iwakura, Gordon D Brown

**Affiliations:** 1 Aberdeen Fungal Group, Section of Immunity and Infection, Institute of Medical Sciences, University of AberdeenAberdeen, Scotland, UK; 2 Division of Molecular Immunology, Medical Mycology Research Centre, Chiba University1-8-1 Inohana, Chuo-Ku, Chiba, Japan; 3 Institute of Medical Sciences, Centre for Experimental Medicine and Systems Biology, University of TokyoMinato Ku, Tokyo, Japan

**Keywords:** Antifungal immunity, Dectin-1, Dectin-2, Mincle

## Abstract

Fungal infections are affecting an increasing number of people, and the failure of current therapies in treating systemic infection has resulted in an unacceptably high mortality rate. It is therefore of importance that we understand immune mechanisms operating during fungal infections, in order to facilitate development of adjunctive immunotherapies for the treatment of these diseases. C-type lectin receptors (CLRs) are pattern recognition receptors (PRRs) that are critical for immune responses to fungi. Many of these receptors are coupled to Syk kinase, which allows these receptors to signal via CARD9 leading to NF-κB activation, which in turn contributes to the induction of both innate and adaptive immunity. Dectin-1, Dectin-2 and Mincle are all CLRs that share this common signalling mechanism and have been shown to play key roles in antifungal immunity. This review aims to update existing paradigms and summarise the most recent findings on these CLRs, their signal transduction mechanisms and the collaborations between these CLRs and other PRRs.

## Introduction

C-type lectin receptors (CLRs) are pattern recognition receptors (PRRs) with C-type lectin-like domains (CTLD) in their extracellular region. Some CLR family members recognise pathogen-associated molecular patterns (PAMPs), whereas other members recognise endogenous ligands. Similar to other PRRs, such as the Toll-like receptors (TLRs), CLRs are involved in host defence against pathogenic infection; however, in contrast to TLRs, which recognise various PAMPs such as lipopolysaccharides, proteoglycans and nucleic acids, CLRs mostly recognise carbohydrates on pathogens.

**Figure 1:**
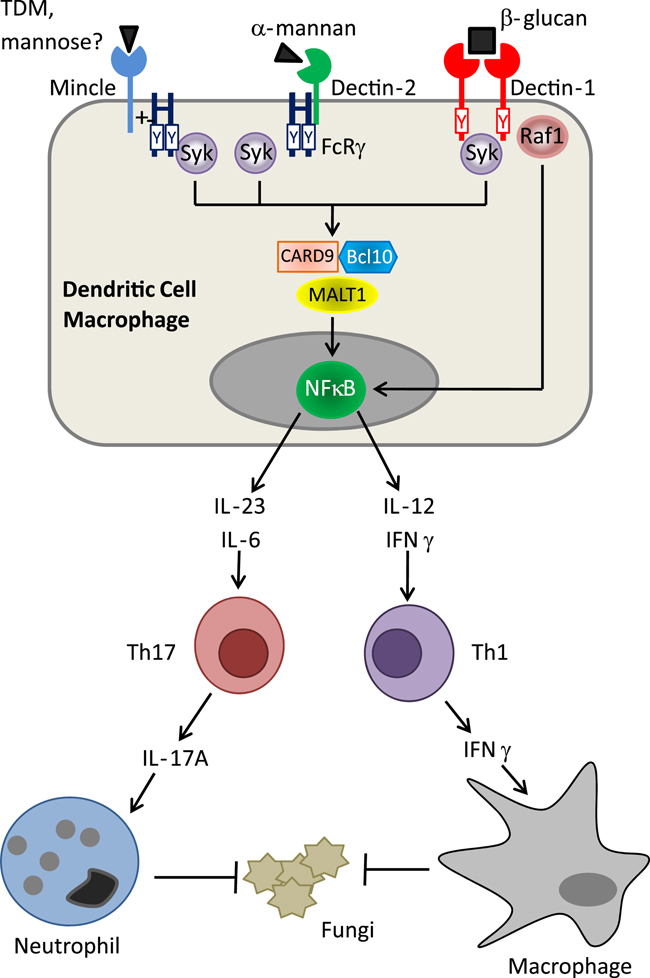
Signal transduction pathways and outcomes of Syk/CARD9-coupled receptor signalling in antifungal immunity. Dectin-1/2 and Mincle all signal via Syk kinase, which associates to either the ITAM-like motif of Dectin-1 (shown by red Y in white boxes) or the ITAM on FcRγ (denoted by dark blue Y in white boxes) that associates with Dectin-2 and Mincle. Syk signalling results in a complex of CARD9, BCL10 and MALT1 that ultimately leads to the activation of transcription factors, including NF-κB. These transcription factors mediate the translation of key cytokines that help to promote Th1/Th17 differentiation, which in turn stimulates antifungal mechanisms from innate cells such as neutrophils and macrophages.

Fungal cell walls contain multiple types of carbohydrates, such as mannans, β-glucans and chitin. Upon recognition of these carbohydrates by PRRs, the host innate immune system becomes activated and antifungal immune mechanisms are initiated. Significant progress has been made in the identification of recognition receptors and their downstream signal transduction; however there are many aspects that remain poorly understood. Recently, we reported that prototypal CLRs, Dectin-1 (gene symbol *Clec7a*) and Dectin-2 (*Clec4n*), are the specific receptors for fungal β-glucans and α-mannans, respectively 1–3. Mincle (*Clec4e* or *Clecsf9*) is another recently discovered CLR that is less well characterised, but nonetheless plays a role in antifungal immunity 4. These receptors induce cytokines that drive inflammation and adaptive immunity to protect the host from infection. These critical actions are dependent on the activation of a common signalling pathway involving Syk kinase, CARD9 and NF-κB (Fig. 1) in DC and macrophages 5. In this review, we summarise the function of these receptors in host defence against fungal infections, their signal transduction mechanisms, and the collaborative responses between these CLRs and other PRRs.

## Dectin-1

Dectin-1 is a predominant PRR involved in antifungal immunity and is primarily expressed by myeloid cells. The PAMP recognised by Dectin-1 is β-glucan, although Dectin-1 has also been shown to recognise unidentified bacterial and endogenous ligands. Studies with Dectin-1-deficient mice have demonstrated Dectin-1's protective role during infection with *Candida albicans* 6, *Aspergillus fumigatus* 7 and *Pneumocystis carinii* 8; however, these studies have also highlighted some inconsistencies with regards to Dectin-1's role in the control of infection with *C. albicans*, with both redundant and non-redundant roles observed 6, 8. Our unpublished data suggest that these inconsistencies are due to differences in the fungal strains used in each study (Marakalala and Brown, unpublished data). A polymorphism in the gene for human Dectin-1, Y238X, which results in an early stop codon and leads to abrogated Dectin-1 expression, has been identified in patients with recurrent mucosal candidiasis. Cells from these patients had poor cytokine responses to *C. albicans* and β-glucan in vitro, indicating that these responses also depend on Dectin-1 in humans 9.

Several studies have helped establish the role of Dectin-1 in both innate and adaptive immunity, and have highlighted how the dependence on Dectin-1 may vary with external conditions, such as host species, the fungal pathogen in question and host cell type. Various isoforms of Dectin-1 have been identified in humans and mice 10, and differences in the expression pattern of these isoforms have also been observed between these two species 11. Dectin-1 is important for the recognition and phagocytosis of fungi, although this function is redundant, as uptake can also be restored by opsonisation 6 and human cells deficient in Dectin-1 have normal fungal phagocytosis 9. Most studies suggest that Dectin-1 is the major initiator of the respiratory burst regardless of fungal species, although this function can be rescued by opsonisation 6. Interestingly, some antifungal actions of Dectin-1 appear to only be redundant with certain pathogens. Saijo et al. 8 demonstrated that TNF-α and IL-12 production were not inhibited in Dectin-1-deficient mice infected with *P. carinii*, while other studies looking at *C. albicans* and *A. fumigatus* showed a strong dependence on Dectin-1 for these inflammatory cytokines 6, 7. Likewise, differences in the prominence of Dectin-1 are found between cell types, as macrophages deficient in Dectin-1 are severely affected in their cytokine production while DCs are only modestly affected 6.

The induction of adaptive immunity by Dectin-1 is currently an intense area of research as the adaptive responses governing antifungal immunity are vital to efficient resolution of fungal infections. Dectin-1 has been shown to induce humoral responses 12 and stimulate cytotoxic T cells in both mice 13 and humans 14. Dectin-1 activation has also been linked to the induction of CD4^+^ T-cell responses as Dectin-1 agonists have been shown to activate DCs and promote Th1/Th17 differentiation 15. In addition, Osorio et al. 16 showed that Dectin-1-activated DCs could instruct Treg (CD25^+^Foxp3^+^) to express IL-17. While it is well accepted that Dectin-1 is able to induce Th17 responses, it is controversial whether Dectin-1 promotes these responses during a fungal infection. Dectin-1 was shown not to be required for IL-17 production in mice infected with *C. albicans* 15. In contrast, humans deficient in Dectin-1 appear to have diminished Th17 responses and this may be linked to their susceptibility to mucosal candidiasis 9; however, this discrepancy could reflect limitations in current studies, or be another species difference that requires clarification.

## Dectin-2

Dectin-2 is expressed by DCs and macrophages 17. This molecule has a CRD containing an EPN motif, which is a Ca^2+^-dependent mannose-binding amino acid sequence, and binds high mannose-type carbohydrates 18–20. Accordingly, Dectin-2 was recently shown to be the functional receptor for α-mannans 3. Dectin-2 has also been implicated in anti-bacterial immunity 21 and allergy 22. Dectin-2 was recently shown to be essential in mediating protection against *C. albicans*, as survival was significantly decreased in Dectin-2-deficient mice when infected with various strains of *C. albicans* 3. Interestingly, two recent studies observed a difference in the ability to induce cytokines between *C. albicans* yeast and hyphae. Soluble Dectin-2 seems to preferentially bind the hyphal form of *C. albicans* 20, and accordingly hyphal forms of the fungus strongly induced cytokines from BMDCs. In contrast, Dectin-2-deficient cells show a substantial reduction in cytokine production when stimulated with yeasts and only a modest one with hyphae, suggesting several other receptors are likely involved in hyphal recognition 3, 23. Studies using a specific blocking mAb against Dectin-2 23 and genetically deficient mice 3 have illustrated how Dectin-2 is involved with cytokine production and adaptive immunity in response to *C. albicans*. Abrogating Dectin-2 in either fashion resulted in impaired *C. albicans*-induced cytokine production from BMDCs, and this presumably impacted on the ability of the DCs to drive Th1/Th17 differentiation, which was also defective in the Dectin-2-deficient cells/mice 3, 23. This result may explain why Dectin-1 deficiency does not alter IL-17 production.

In the field of antifungal immunity, Th17 responses have been of great interest recently, as it remains relatively unclear whether Th17 responses are required for effective antifungal immunity. The importance of IL-17 in *C. albicans* infections has been demonstrated using mice deficient in IL-17RA or IL-23p19 24, 25. IL-17A-deficient mice, but not IL-17F-deficient mice, are more susceptible to systemic infection with *C. albicans*; it has been proposed that these differences are due to the functioning of these cytokines between different immunological settings 3. A link between fungal susceptibility and Th17 responses has also been made in humans – several human mutations resulting in immune disorders (e.g. APECED and HIES) that are characterised by abrogated Th17 responses are associated with chronic mucocutaneous candidiasis. 26–28. While these studies make a strong case for the beneficial effect of Th17 responses during fungal infections, other studies have presented convincing data that those Th17 responses may well be damaging in antifungal immunity, by exacerbating inflammatory-mediated damage 29. It has also been argued that Th17 immunity may be required only to control fungal infections at specific sites, such as the oral mucosa 30.

## Mincle

Mincle is a member of the Dectin-2 family of CLRs, and is a non-phagocytic receptor; its expression on macrophages is controlled by inflammatory stimuli. The fungal ligand for Mincle is not well defined; however, recent studies using microarrays and mutational studies have strongly suggested that the ligand is α-mannose 31. Mincle can recognise an endogenous ligand, SAP130 32, and has been implicated in anti-mycobacterial immunity due to its recognition of a cell wall component (see below). Studies using gene-deficient mice have demonstrated the importance of Mincle in antifungal immunity; these studies revealed that Mincle was important for cytokine (particularly TNF-α and IL-10) and chemokine production during fungal infection 31, 33. A role in phagocytosis was suggested, as Mincle was recruited to the phagosome around yeast particles, although both a blocking antibody and experiments with *Clec4e*^−/−^ macrophages showed no defect in the phagocytosis of *C. albicans*. Nonetheless, mice deficient in Mincle had substantially higher fungal burdens than WT mice when infected with *C. albicans,* indicating that Mincle was required for resolving these infections 33; however, this study only looked at an early time point (5 days following infection) and did not compare the long-term survival of knockout and WT mice.

Another study by Yamasaki et al. 31 used cell reporter systems to investigate which fungal species were recognised by Mincle. Their results show that Mincle only specifically recognised *Malassezia* species and not others tested 31. The authors also show an intimate involvement of Mincle with anti-*Malassezia* responses. No current mouse model exists for an in vivo *Malassezia* infection; hence, the authors used an alternative system whereby the fungus was injected intraperitoneally. This approach generated neutrophil infiltration and an upregulation in IL-6 and TNF in the peritoneal cavity, which was lost in Mincle-deficient mice 31. In contrast to the previous study, Yamasaki et al. 31 showed no connection between Mincle and *C. albicans* immunity; however, there were differences in the strains used in each study and hence Mincle, as with Dectin-1, may operate a similar paradigm with respect to *C. albicans*-dependent and -independent strains.

Although Mincle has not been shown to induce adaptive immunity during antifungal responses, this has been observed during anti-mycobacterial responses. Mincle binds to a key component of mycobacteria, trehalose-6,6′-dimycolate (TDM, also known as cord factor), as well as a synthetic derivative (trehalose-6,6-dibehenate, TDB). These compounds have been investigated for their adjuvant activity and it was recently shown that Mincle was the key receptor involved in the induction of cytokines and nitric oxide in response to TDM/TDB 34. Interestingly, this interaction with TDM and its derivative was recently shown by Schoenen et al. 35 to be involved in the induction of Th1/17 responses, as the adjuvant effect of TDM and Th1/Th17 responses were lost in Mincle-deficient mice. These downstream actions have not yet been strongly linked to antifungal immunity; however, research into Mincle is at an early stage and further work using more in-depth studies of in vivo infection models with other fungal species will inevitably help clarify the role of Mincle in antifungal immunity.

## Syk-CARD9 signal transduction

Dectin-1 has an immunoreceptor tyrosine-based activation motif (ITAM)-like in its cytoplasmic domain. An ITAM is a well-characterised motif consisting of a repeated sequence (YXXI/L) that upon ligand binding become phosphorylated by Src family kinases and recruit SH2-containing tyrosine kinases (e.g. Syk). The ITAM-like motif of Dectin-1 resembles this sequence although, unexpectedly, signalling can occur when only the membrane proximal repeat is phosphorylated. Syk however, requires two tyrosine (Y) residues for recruitment hence it is hypothesised that Dectin-1 may form a dimer structure with Syk, following activation 11, 36 (Fig. 1). Dectin-1-dependent cytokine production, MAPK activation, and NF-κB activation, are all inhibited by Syk deficiency or Syk inhibitors, further suggesting that Syk is essential for Dectin-1 signalling. Importantly, however, not all Dectin-1 signalling is dependent on Syk; for example, phagocytosis of zymosan initiated by Dectin-1 in macrophages is Syk-independent 37. This has led to the identification of several other pathways emanating from Dectin-1. For example, calcium-mediated pathways induce IL-10 and the respiratory burst 38, while activation of the Nalp3 inflammasome, another intracellular PRR, is important for caspase-1 activation and IL-1β release 39. Furthermore, Dectin-1 also activates the Raf-1-dependent signalling pathway 40, 41. This Raf-1 pathway is Syk-independent but integrates with the Syk-dependent pathway at the level of NF-κB activation and contributes to enhance Th1 and Th17-mediated responses (Fig. 1).

Dectin-2 and Mincle have no known signalling motifs in their intracellular regions; however, through the association with the FcRγ chain, a membrane-associated adaptor protein that contains a traditional ITAM sequence, these receptors are able to activate Syk indirectly. Dectin-2 and Mincle associate with FcRγ through slightly different protein–protein interactions; Dectin-2 appears to use its intracellular region proximal to the membrane while Mincle forms a salt bridge through a positively charged arginine residue in its transmembrane region 20 (Fig. 1). FcRγ has been shown to be essential for Dectin-2 surface expression and signalling 20, 23. Similar experiments with Mincle show a selective association with FcRγ over other adaptors (e.g. DAP12) and this was evident in both mouse and human cells 42.

Syk represents a common point in the signalling pathways of Dectin-1, Dectin-2 and Mincle. Downstream of Syk activation, an adaptor protein known as CARD9 forms a trimolecular complex with BCL10 and MALT1, which is required for signalling from these receptors (Fig. 1). The steps linking Syk and CARD9 are currently undefined. CARD9 has a coiled-coil region at the C-terminus and a CARD domain in the N-terminus; however, unlike other CARD-containing membrane-associated guanylate kinase (CARMA) family proteins, CARD9 lacks a PDZ domain that is necessary for association with the plasma membrane 43. The use of *Card9*^−/−^ mice revealed that CARD9 is essential for the cytokine and chemokine production induced by Dectin-1, Dectin-2 and Mincle 42. The crucial role of CARD9 in the signalling of these receptors may be linked to a requirement for CARD9 in effective antifungal responses. *Card9*^−/−^ mice are more susceptible to *C. albicans* 3, while humans carrying *Card9* mutations also have an increased susceptibility to life-threatening infections with *C. albicans* 44. While CARD9 is clearly implicated in the protection against *Candida* infections, it has yet to be shown to what extent immunity to other pathogenic fungi is CARD9-dependent.

## Collaborative responses between PRRs

Knockout gene studies have highlighted that most PRRs demonstrate some degree of redundancy in certain roles. For example, most antifungal responses have been shown to be absolutely dependent on Syk kinase and CARD9 rather than on a single receptor 23. This suggests that several different receptors are needed in antifungal immunity. Indeed, several groups have identified key relationships between PRRs that are used to initiate and sustain effective antifungal immunity.

A well-known collaboration in antifungal immunity is that between Dectin-1 and TLR2. Although known for some time, the exact molecular details underlying their synergistic nature remain unknown; however, it has been established that this synergism is dependent on Syk 45. The synergy observed between TLR2 and Dectin-1 is most pronounced during inflammatory cytokine production and NF-κB activation 46. For example, stimulation of macrophages with Dectin-1 and TLR2 agonists has a synergistic effect on the production of TNF, IL-6 and IL-23, but simultaneously downregulated IL-12 – responses that are thought to be important for shaping the type of adaptive response 45. Another example is IL-1β, which is essential for antifungal immunity, and the use of double knockout mice (*Tlr2*^−/−^*Clec7a*^−/−^) has demonstrated the requirement of both these receptors in the production of IL-1β 39. The role of the TLR2/Dectin-1 collaboration however, is less well defined in other areas of immunity. For example, phagocytosis is Dectin-1-dependent, and although the TLRs have been demonstrated to regulate phagosomal maturation in certain experimental settings, TLR2^−/−^ macrophages showed no defect in their internalisation of zymosan 46. In contrast, TLR2 may enhance the respiratory burst initiated by Dectin-1, by priming the ability of macrophages to carry out this function 46. Interestingly, the synergistic relationship is not restricted to TLR2, and Dectin-1 has been shown to collaborate with TLR1 47, −4, −5, −7 and −9 48.

Another important synergistic relationship, at least during *C. albicans* infection, was found between Dectin-1 and Dectin-2. Robinson et al. 23 used a blocking mAb against Dectin-2 to treat WT and Dectin-1-deficient DCs. Stimulation of these DCs with heat-killed *C. albicans* or zymosan revealed an almost complete abolishment in cytokine production when both of these CLRs were knocked out. This result was reminiscent of those observed with Syk-deficient cells and therefore it has been proposed that all Syk-dependent responses to *Candida* may depend on Dectin-1 and Dectin-2. These experiments were also reproducible in vivo, as Dectin-1-deficient mice that received the Dectin-2-blocking antibody had a significantly decreased Th1 response during systemic candidiasis infection; however, Th17 responses were only dependent on Dectin-2, and not Dectin-1, suggesting that Dectin-2 is the main receptor involved in driving these responses during *C. albicans* infection 23.

While relationships between pairs of PRRs are becoming well defined, it is likely that antifungal immunity requires input from complex cross-talk of multiple PRRs. This is an aspect of antifungal immunity that is unclear; however, a study by Netea et al. 49 was able to give an initial insight into the integrative nature of antifungal responses. Using a variety of *C. albicans* cell wall mutants and specific ligand blocking experiments, the authors demonstrated how multiple receptors, including Dectin-1, TLRs and the mannose receptor (MR), were required for the recognition of invasive fungal particles, which in turn was essential for efficient clearance of the infection 49. Further research should aim to clarify the relationships between PRRs and how these are used in immunity. It shall be interesting to see whether different factors, such as fungal pathogen and cell type, impact on integrative responses differently.

## Concluding remarks

There is a growing appreciation for the importance of CLRs in antifungal immunity. Dectin-1/2 and Mincle all play vital roles as outlined here, but are likely to be carried out in collaboration with other receptors. Their role may, however, vary depending on the pathogenic fungi and host species in question, and these types of differences have already been identified for some of these receptors, such as with Dectin-1. While significant progress in our understanding of these receptors has been made, several questions remain. For example, is of interest to investigate the roles of Dectin-1/2 in the differentiation of Th17 in humans, and to fully understand the functional roles of IL-17 and Th17 responses in the host defence against fungal infections. Additionally, it is not well understood how several CLRs that signal through a common pathway can give rise to diverse responses. Future investigations into these and other receptors should facilitate the development of novel antifungal treatments and vaccines, and hopefully reduce mortality rates associated with fungal infections.

## Abbreviations

CLR: C-type lectin receptor
TDM: trehalose-6, 6′-dimycolate
